# A large invasive chondroblastoma on the temporomandibular joint and external auditory canal: a case report and literature review

**DOI:** 10.1186/s40902-021-00313-7

**Published:** 2021-07-14

**Authors:** Heeyeon Bae, Dong-mok Ryu, Hyung Kyung Kim, Sung-ok Hong, Hyen Woo Lee, Youngjin Shin, Yu-jin Jee

**Affiliations:** 1 Department of Oral and Maxillofacial Surgery, Kyung Hee University Dental Hospital at Gang-dong, #892 Dongnam-ro, Gangdong-gu, Seoul, 05278 Republic of Korea; 2 Department of Oral and Maxillofacial Surgery, College of Dentistry, School of Dentistry, Kyung Hee University, Seoul, Republic of Korea; 3 Department of Pathology, Kyung Hee University Hospital at Gang-dong, Seoul, Republic of Korea

**Keywords:** Chondroblastoma, Temporomandibular joint, Pre-auricular approach, Temporoparietal fascia flap, Inguinal fat graft, Multidisciplinary approach

## Abstract

**Background:**

Chondroblastomas, which account for approximately 1% of all bone tumors, typically occur in long bones, such as the femur, humerus, and tibia. However, in extremely rare cases, they may also occur in the craniofacial region where the tumor is often found in the squamous portion of the temporomandibular joint (TMJ) and in the temporal bone.

**Case presentation:**

This case report describes a large chondroblastoma (diameter, approximately 37 mm) that occurred in the TMJ. The tumor was sufficiently aggressive to destroy the TMJ, mandibular condyle neck, external auditory canal (EAC), mandibular fossa of the temporal bone, and facial nerve. The tumor was completely excised using a pre-auricular approach. The EAC and surgical defect were successfully reconstructed using a temporoparietal fascia flap (TPFF) and an inguinal free fat graft. There was no local tumor recurrence at the 18-month follow-up visits. However, the patient developed sensory neural hearing loss, and his eyebrow paralysis worsened, eventually requiring plastic surgery.

**Conclusion:**

Large, invasive chondroblastomas of the TMJ can be completely removed through a pre-auricular approach, and the resulting surgical defect can be reconstructed using TPFF and free fat grafts. However, preoperative evaluation of the facial nerve and auditory function is necessary. Therefore, a multidisciplinary approach is essential.

## Background

Chondroblastomas are cartilaginous neoplasms that account for approximately 5% of all benign tumors [1] and approximately 1% of all bone tumors [2]. Moreover, 70% of these neoplasms are reported in long bones, such as the femur, proximal humerus, and proximal tibia [3]. Only 7% of chondroblastomas occur in the craniofacial region, and only 70 cases of chondroblastoma of the temporal bone were reported until 2011 [4, 5]. In the craniofacial region, chondroblastomas typically occur in the squamous portion of the temporal bone and in the temporomandibular joint (TMJ). This is because the bones comprising the skull base develop from cartilage cells [6]. Most chondroblastomas of the long bones occur during the teenage years, but in the craniofacial region, they are known to occur between 30 and 40 years of age [7]. The ratio of male-to-female prevalence is approximately 2:1 [8].

The chondroblastoma reported in this paper was uncommonly large (diameter, approximately 37 mm), occurred in the craniofacial region, and involved the TMJ, temporal bone, and neck of the mandibular condyle. Although it was a benign tumor, it was aggressive and destroyed adjacent anatomical structures. We report this case because it illustrates a method for the successful excision of this type of tumor, and the successful reconstruction of the resulting large defect.

## Case presentation

### History and physical examination

A 52-year-old man was referred to our department by an otolaryngologist for an evaluation of a mass that was detected on his TMJ, using computed tomography (CT) and magnetic resonance imaging (MRI). The patient had an operative history that included the repair of a hernia and a ruptured cruciate ligament, but with no other specific medical history. The patient’s chief complaints were swelling and asymmetry of his right TMJ area, which occurred approximately 2 weeks before the first visit. He also experienced tenderness in the TMJ area upon palpation, but without spontaneous pain. In addition, paralysis of the right eyebrow was observed, suggesting that the mass had damaged the temporal branch of the facial nerve. The detected mass was then fixed and confirmed. The EAC swelling was confirmed by the otolaryngologist. The mandible deviated to the right at the maximum intercuspal position. The maximum mouth opening was limited to 30 mm. The patient had a history of TMJ dislocation approximately 25 years earlier.

### Imaging findings

**Fig. 1:**
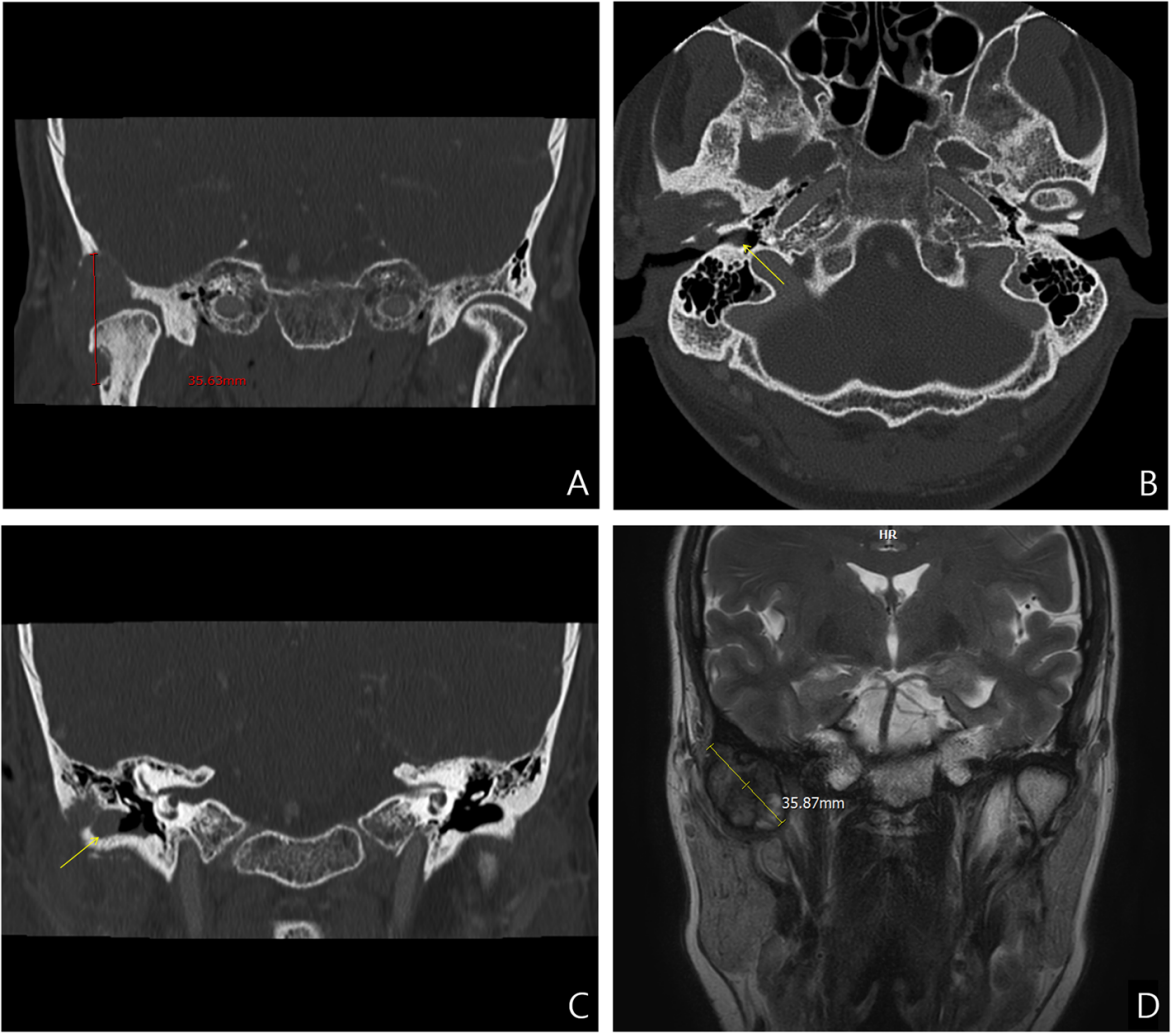
Preoperative computed tomography **A** coronal view shows erosion of the skull base temporal bone and the moth-eaten appearance of the right mandible neck. The mild thickening of the tympanic membrane (**B**, arrow) and fluid collection in the external auditory canal (**C**, arrow) were tentatively diagnosed as external otitis due to an error in the interpretation of the images. Preoperative magnetic resonance imaging **D** coronal view shows an inhomogeneous, multi-lobulated mass with an intermediate signal

CT clearly showed a mass of eccentric soft tissue (approximately 37 × 28 × 28 mm) surrounding the right TMJ and extending into the right pterygoid space; the tissue had also eroded the temporal bone of the skull base. In addition, bone destruction (having the appearance of being moth eaten) was observed in the right mandibular condyle neck (Fig. 1A). The CT scan showed mild thickening of the tympanic membrane (Fig. 1B, arrow) and fluid collection in the EAC (Fig. 1C, arrow). These findings were tentatively diagnosed as external otitis by the radiologist. T2-weighted MRI showed that the tumor was a well-marginated, ovoid-shaped mass with multiple inhomogeneous lobes; the tumor had an intermediate signal similar to that of the gray matter. The deeper areas of the mass showed bright signals, such as those found in the central necrosis of the mass (Fig. 1D).

### Surgical procedure

**Fig. 2:**
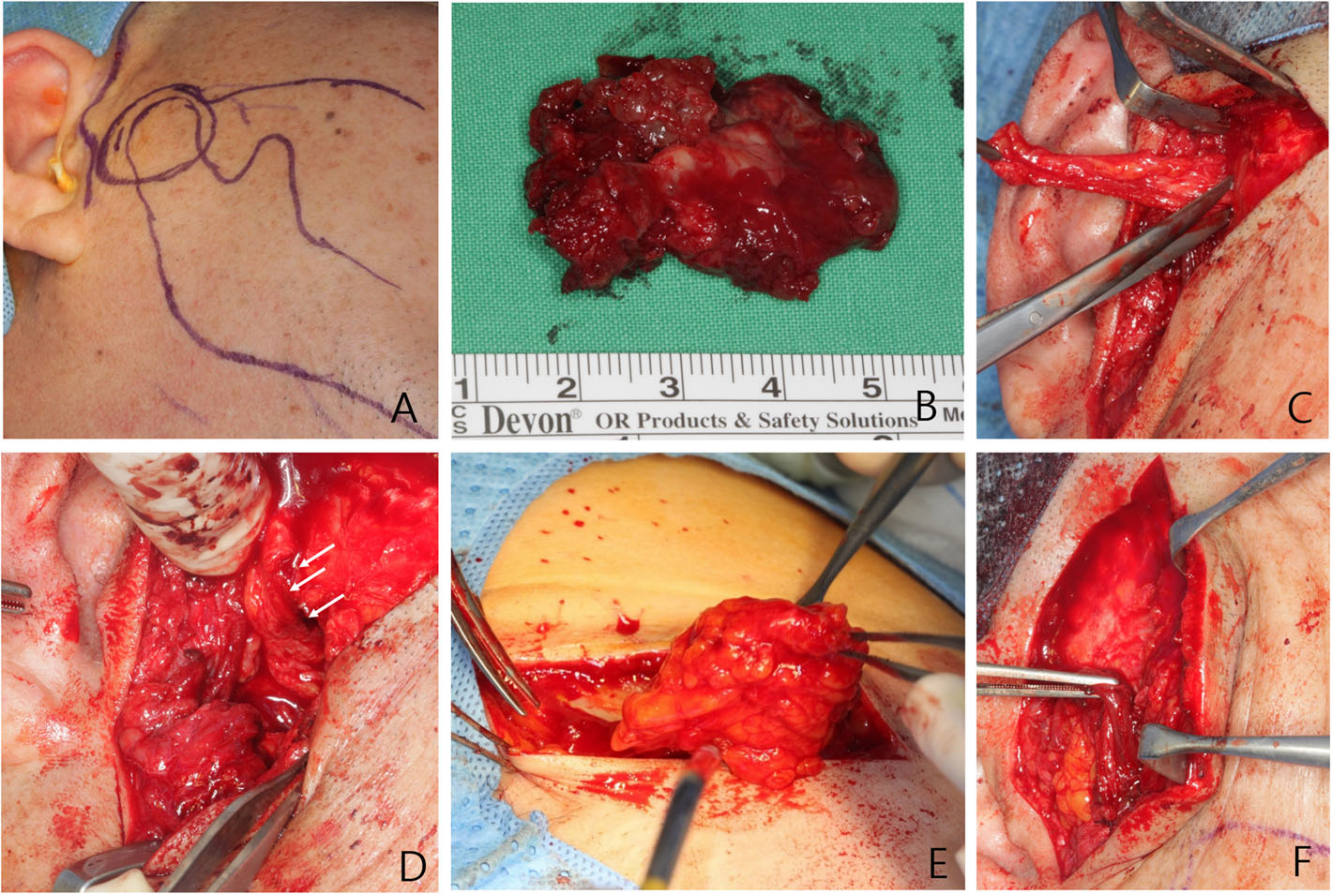
**A** The pre-auricular approach, using a hockey-stick incision, is shown. The incision extends anterosuperiorly. **B** The completely dissected and removed tumor is shown. The largest tumor dimension is 37 mm. **C** The temporal fascia flap is harvested. **D** A defect in the anterior wall of the external auditory canal is covered using a temporal fascia flap (arrow). **E** An inguinal free fat graft is shown. **F** The inguinal fat graft is covered by a pedicled temporoparietal fascia flap (TPFF)

Under general anesthesia, complete surgical excision was performed using a pre-auricular approach. Local anesthesia (2% lidocaine with 1:100,000 epinephrine) was injected into the temporal and pre-auricular skin regions to reduce intraoperative bleeding and postoperative pain. Access was obtained via an extended pre-auricular (hockey-stick) incision, with an oblique anterosuperior extension into the hair-covered temporal region (Fig. 2A). To reach the main mass, blunt dissection was performed up to the temporal fascia, and bleeding control was achieved by ligating the superficial temporal artery and vein. The tumor was carefully separated from the condyle and the mandibular fossa of the temporal bone to achieve its complete removal (Fig. 2B). The tumor had invaded the anterior articular disc of the TMJ, necessitating partial resection of half of the disc. After tumor excision, peripheral ostectomy of the neck of the condyle and mandibular fossa of the temporal bone was performed using round burs. The neurosurgeon confirmed that the bony erosion of the mandibular fossa was severe enough to expose the dura mater of the brain, but no perforation of the dura was observed. As the probability of cerebrospinal fluid leakage was extremely low, no further surgical procedures were required. The tumor was observed to have also destroyed the anterior wall of the right EAC. An otolaryngologist confirmed that the tympanic membrane was intact. An absorbable gelatinous foam packing was applied in front of the tympanic membrane to maintain the remaining EAC. The EAC defect was covered using a temporal fascia flap pedicled to the deep temporal fascia layer (Figs. 2C and D).

After complete removal of the tumor, the remaining defect was extensive. If the defect had remained, there would have been a substantial risk of facial depression and secondary infection due to the accumulation of fluid and blood in the dead space. To compensate for the extensive volume loss, a free fat graft from the right inguinal region (Fig. 2E) was performed on the right TMJ. The transplanted inguinal free fat graft was connected to the remaining articular disc and covered by the pedicled TPFF (Fig. 2F).

### Pathologic examination

**Fig. 3:**
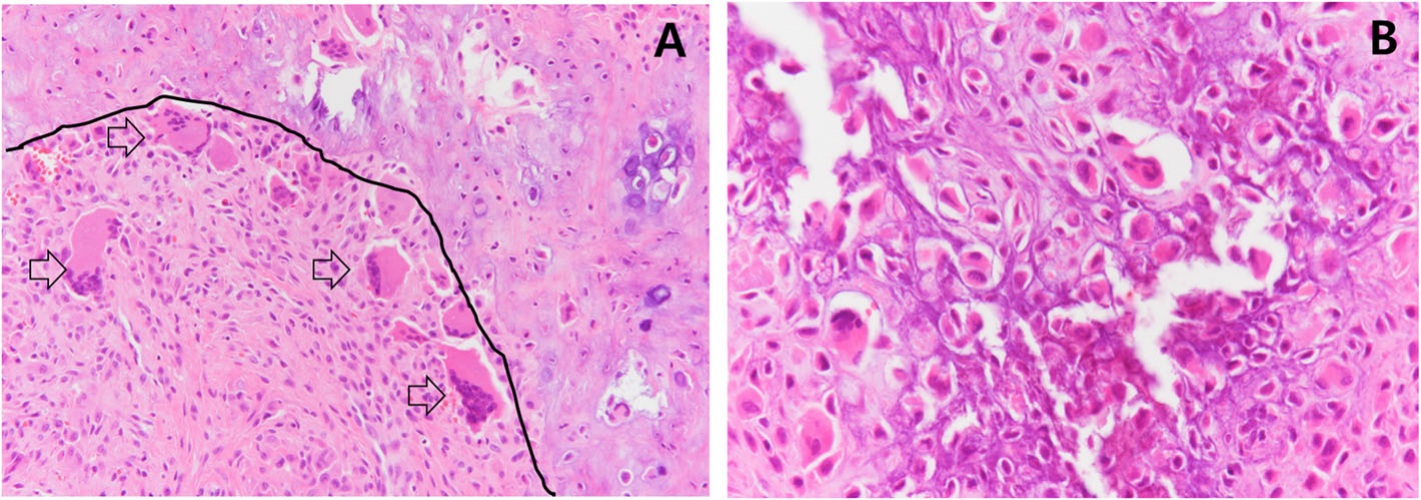
**A** The tumor is composed of sheets of mononuclear cells (below the line) admixed with occasional multinuclear cells (arrow); hematoxylin and eosin stain, 200×. The eosinophilic and basophilic chondroid matrix (above the line) is intermingled. **B** The degenerative area shows perinuclear lace-like calcification (hematoxylin and eosin stain, 400×)

Pathologic examination revealed a cellular tumor composed of multifocal chondroid tissue (Fig. 3A, above the line) and sheets of tumor cells (Fig. 3A, below the line). Ovoid to polygonal multinuclear cells (Fig. 3A, arrow) comprised the tumor tissue, each having occasional nuclear grooves and eosinophilic cytoplasms. Osteoclast-like giant cells and pericellular lace-like calcifications (Fig. 3B) were also identified. The pathologic examination confirmed the chondroblastoma diagnosis.

### Postoperative course

**Fig. 4:**
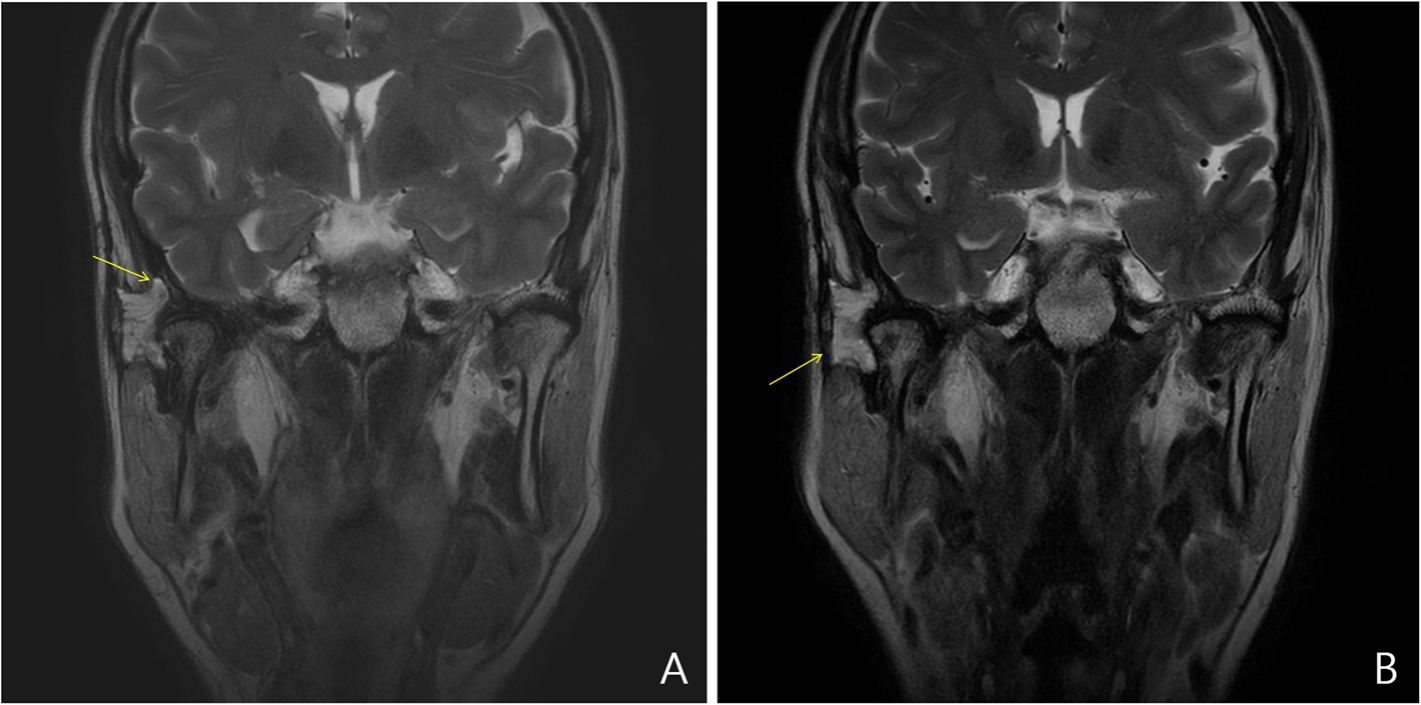
Magnetic resonance imaging (**A**) coronal scan, 7 months postoperatively, shows the stable inguinal fat pad graft covering the temporal bone defect (arrow) and do not indicate chondroblastoma recurrence. At the 18-month follow-up visit, the magnetic resonance imaging (**B**) coronal view fails to reveal newly developed, abnormally enhanced lesions or restricted diffusion into the operation bed (arrow). There is no evidence of local recurrence

One month after the operation, the patient had not developed trismus, but the paralysis of the right eyebrow remained. In addition, the patient complained of tinnitus in his right ear. After 7 months, a follow-up MRI revealed resolved muscle edema at the right masseter and pterygoid. In addition, stable coverage of the defect in the temporal bone was achieved with the inguinal free fat graft (Fig. 4A, arrow), and there was no evidence of chondroblastoma recurrence. The swelling and asymmetry of the right TMJ area decreased, and the maximum mouth opening range was maintained at 28 mm. Postoperatively, the paralysis of the patient’s right eyebrow worsened, and right eyelid ptosis developed; supra-brow excision and blepharoplasty were required, after consulting with plastic surgeons, 8 months after the operation. At the 18-month follow-up, the patient demonstrated normal occlusion and no masticatory disturbance. An MRI did not reveal any newly developed, abnormally enhanced lesions or any evidence of local recurrence (Fig. 4B).

## Discussion

### Literature review

We reviewed the cases of 11 individuals with craniofacial chondroblastoma who were described in 11 previously published papers (Table 1 [9–19]). Including our case, the review included five male and seven female patients (median age, 44.5 years; range, 27–66 years). In eight cases, the radiographic examinations revealed destroyed the TMJ and mandibular condyle; temporal bones were affected in five cases. In four cases, the chondroblastomas affected the anatomical structures of the middle ear, producing hearing loss. In some cases, the clivus and sphenoid sinus were destroyed. In the earliest (1971) included case, curettage was used to remove the lesion, but it recurred and remained. Therefore, a second operation was required. In addition, there was a tendency for treatments to involve complete excisions. The pre-auricular approach was used in five cases. Three cases used the endoscopy and rhinoscopy, and one case employed the middle cranial fossa approach. To reduce damage to the anatomical structures around the TMJ and minimize postoperative dysfunction, the middle cranial fossa approach has also been used in recent years. The advantage of this approach is that it is easy to secure a surgical field and access the dura mater [19]. In addition, some cases used the transpetrosal-transzygomatic approach, the modified auriculotemporal approach using a U-shaped incision, and the retro-auricular approach. Reconstruction using the temporalis muscle flap was performed in two cases, and there were two cases that involved the use of the temporal fascia layer and one that used the nasoseptal flap. In two cases, reconstruction of the surgical defect was performed using a fat graft. In 11 cases, the treatment resolved the chondroblastoma.

**Table 1:** Summary of characteristics for patients with craniofacial chondroblastomas (from 11 reports)

Author (year)	Age/ sex	Site	Size (mm)	Symptoms	Surgical methods	Reconstruction	Recurrence (follow-up period)
Cares et al. (1971) [9]	30/F	• Fronto-zygomatic area • Anterior superior EAC	• Not described	• Temporal area swelling • Plugged ear sensation • Conductive hearing loss	• 1st OP: curettage • 2nd OP: excision (1 month after 1st OP)	• Not described	No recurrence (2 years)
Longo et al. (1999) [10]	27/M	• TMJ • Mandibular condyle • Temporal bone	• Not described	• TMJ swelling • Mouth opening deviation	• Subtotal excision (extended pre-auricular, temporal approach)	• Not described	No recurrence (1 year)
Watanabe et al. (1999) [11]	43/F	• TMJ • Temporal bone • Middle ear • EAC	• 15 x 20	• Chronic otitis media • Conductive hearing loss	• Complete excision (retro-auricular approach)	• Surgical defect (Temporal muscle flap)	No recurrence (4 years)
Toro et al. (2005) [12]	57/F	• TMJ • Mandibular condyle	• 20 x 20	• TMJ swelling • Mouth opening deviation • TMJ clicking	• Complete excision (pre-auricular approach to deep sub-fascia)	• Not described	No recurrence (1 year)
Bian et al. (2005) [13]	38/M	• Temporal bone • Zygomatic arch	• 30	• Temporal area swelling • Conductive hearing loss	• Complete excision (middle cranial fossa approach)	• Not described	No recurrence (1.25 years)
Kim et al. (2015) [14]	49/F	• Mandibular condyle	• 20	• TMJ swelling • Pre-auricular pain, swelling • Trismus	• Complete excision (pre-auricular approach)	• Not described	No recurrence (8 years)
Liu et al. (2015) [15]	27/F	• Clivus • Carotid canal	• 28 x 20 x 19	• Headache • Diplopia • Facial dysesthesias	• Complete excision • Endoscopic endonasal approach	• Surgical defect (nasoseptal mucosal flap)	No recurrence (3 months)
Hiraumi et al. (2016) [16]	64/M	• TMJ • Middle cranial fossa • Superior semicircular canal • Foramen spinosum • Facial nerve • Otic capsule	• Not described	• Vertigo	• Complete excision (transpetrosal-transzygomatic approach)	• Eardrum, EAC (temporal fascia flap) • Surgical defect (abdominal fat)	No recurrence (5 years)
Marano et al. (2019) [17]	46/M	• Mandibular condyle	• 21 x 10 x 17	• TMJ pain, swelling	• Complete excision (pre-auricular approach)	• Not described	No recurrence (1.5 years)
Long et al. (2020) [18]	40/F	• Sphenoid sinus	• 22 x 20	• Dizziness • Headache	• Complete excision • Rhinoscopic surgery	• Not described	Not described
Tomioka et al. (2020) [19]	66/F	• TMJ • Temporal bone • Middle cranial fossa • EAC	• 35 x 25 x 20	• Hearing loss • Mouth opening deviation • TMJ clicking	• Complete excision (modified auriculotemporal approach via U-shaped incision) • Endoscopic surgery	• Parietal bone (temporal muscle flap, titanium mesh plate)	No recurrence (5.5 years)
Present case	52/M	• TMJ • Mandibular condyle • Temporal bone • EAC	• 28 x 28 x 37	• TMJ swelling, asymmetry • TMJ pain on palpation • Mouth opening deviation • Eyebrow paralysis	• Complete excision (pre-auricular approach)	• Anterior wall of the EAC (temporal fascia flap) • Surgical defect (inguinal fat graft, TPFF)	No recurrence (1.5 years)

*TMJ*, temporomandibular joint; *EAC*, external auditory canal; *OP*, operation

When the tumor occurs in the craniofacial region, as in the present case, pain in the pre-auricular region, occlusal abnormalities, trismus, and TMJ clicking have been reported. Further, the patient may also experience a “plugged ear” sensation, hearing loss, dizziness, and otorrhea. Radiographic examinations usually show well-marginated, ovoid-shaped masses with rarefaction. This imaging finding suggests that the tumor is benign, but is insufficient to confirm a differential diagnosis relative to other bone tumors. Therefore, a histological examination is necessary. The characteristic histological findings of a chondroblastoma include the presence of multinucleated giant cells and a chondroid matrix. These cells show immunoreactivity with the s-100 protein, which differentiates a chondroblastoma from a chondrosarcoma or an aneurysmal bone cyst. Unlike our case, chondrosarcoma of the craniofacial region occurs mainly in patients younger than 20 years of age. Radiographically, chondrosarcoma shows malignant characteristics with ill-defined margins. It also shows histologic features, such as cytologic atypia, as, which are not seen in chondroblastoma [20]. An aneurysmal bone cyst is an osteolytic lesion surrounded by connective tissue, that, unlike chondroblastoma, occurs mainly in patients under 30 years of age [21]. Radiographically, aneurysmal bone cysts are characterized by osseous expansion accompanied by a thin peripheral shell of bone. Histological findings include a hemorrhagic cystic space [22]. Considering our patient's age, clinical symptoms, radiographic and histologic characteristics, the likelihood of the lesion being a chondrosarcoma or aneurysmal bone cyst was low. Therefore, we decided not to perform further immunologic analysis, such as the s-100 protein test.

To date, the pre-auricular approach has been considered the gold standard surgical method for accessing the TMJ. The indication for a pre-auricular approach is any operation that requires access to the upper condyle and TMJ. Efforts have been made to minimize incision scars by using the crease in front of the tragus. Among the modified approaches to TMJ, the post-auricular approach has the advantage of hiding the incision line behind the ear, even in patients with tendencies to develop keloids. However, the post-auricular approach to the TMJ cannot avoid transecting the EAC. Therefore, a wide dissection is necessary to prevent ankylosis of the EAC and external otitis [23]. Some authors have extended the post-auricular incision to the cervical region to enable the excision of a TMJ chondroblastoma and have used a neck dissection to diagnose the metastases of tumors [24]. According to a report published in 2009, some mandibular condylectomies have been performed using a modified trans-oral approach. The flap elevation was performed using a buccal mucosa incision, and the coronoid process was cut at the sigmoid notch level to access the condyle. After the condylectomy, the fragment of the coronoid process was realigned and fixed in its original position. We suggest that this approach is a new method for treating TMJ lesions [25].

The most important point of our case is that a very large (approximately 37 mm) chondroblastoma that was affecting the TMJ was successfully removed. The main tumor had aggressive characteristics and had destroyed the mandibular fossa of the temporal bone, but it was delicately removed, avoiding perforation of the dura mater. Additionally, half of the lateral condyle neck was damaged by the tumor, but the condyle head was preserved to the extent possible to preserve the vertical height of the mandibular ramus. Therefore, the patient did not complain of facial asymmetry, trismus, or deviations when opening his mouth. The anterior part of the articular disc was invaded by the main tumor and resected, and an inguinal free fat graft was connected to the remaining posterior part of the articular disc. Although the patient did not experience abnormal occlusion or a masticatory disorder, it was unreasonable to expect significant improvement in mouth opening due to the loss of half of the articular disc.

The main deficiency associated with the diagnosis of this patient was that the region of the EAC that had been invaded by the tumor was incorrectly diagnosed, based on the preoperative CT, as fluid collection due to external otitis. Hence, the destruction of the EAC was not predicted. Due to this tentative diagnosis, a preoperative puretone audiometry (PTA) examination was not performed. Sensorineural hearing loss (SNHL), at 2 kHz, was detected during postoperative PTA, which may have caused the postoperative tinnitus. We could not confirm whether SNHL existed prior to the operation. If we had preoperatively confirmed SNHL, we could have used minimally invasive surgery to avoid worsening the SNHL. Preoperatively, the patient complained of eyebrow muscle paralysis, which might have been expected due to tumors affecting the temporal branch of the facial nerve. Paralysis of the right eyebrow and ptosis of the right eyelid worsened after the operation. This observation suggests that additional facial nerve damage was caused intraoperatively due to electric cauterization or overly aggressive traction.

Complete surgical excision is the most effective treatment for a chondroblastoma. When en bloc excision is not possible, curettage may be considered as a treatment option. The recurrence rate following complete surgical excision is 20%; however, when treated with curettage, there is a 50% probability of recurrence [26]. In our case, the tumor was very large and had aggressive characteristics, prompting the decision to perform a complete surgical excision, with wide margins. This was chosen over curettage to reduce the chance of recurrence.

The reconstruction of damaged craniofacial areas, especially at the cranial base, is a very delicate operation. The TPFF receives its blood supply from the superficial temporal artery and has a high survival rate, even after radiation therapy. In addition, deep and superficial temporal fascia layers have the advantage of being thin, flexible, and adjustable in length; therefore, they are useful for reconstructing skull base defects. The TPFF is used to reconstruct lateral skull base defects and prevent CSF leakage; they are also used to rebuild defects in the ventral skull base by passing the TPFF through the infratemporal fossa. The modified TPFF can be used to reconstruct anterior skull base defects via the supraorbital epidural corridor [27]. The use of a superficial temporal fascia flap to reconstruct a burned ear has been reported [28].

Although most authors insist that the surgical excision of chondroblastomas is the most effective treatment, there is an argument that suggests that invasive methods should be avoided. Among the recently published papers, several cases involving the use of a microscope and an endoscope have been reported. The microsurgical instruments were used to access the main mass by drilling through the tympanic region and the mandibular condyle. Although a safety margin could not be obtained, there was the advantage of preserving the functioning of the cochlea, facial nerve, jugular vein, and TMJ. Recurrence was observed in some of the cases treated in this manner; however, they were well managed using radiotherapy [29].

There is a debate about the role of radiation therapy in chondroblastoma treatment. In one report, radiation therapy helped prevent recurrence after complete resection of the chondroblastoma [30]. Thus, radiation therapy may be considered to reduce the frequency of chondroblastoma recurrence, but it also increases the possibility of malignancy. Therefore, radiotherapy is not essential [31].

## Conclusion

A large, aggressive chondroblastoma affecting the TMJ was successfully removed using a pre-auricular approach. The EAC and resulting surgical defect were reconstructed using TPFF and inguinal fat grafts. Postoperative complications may occur, depending on the degree of tumor invasion into the surrounding anatomical structures, and a close multidisciplinary approach was necessary.

## Data Availability

Not applicable

## Abbreviations

TMJ: Temporomandibular joint
EAC: External auditory canal
TPFF: Temporoparietal fascia flap
CT: Computed tomography
MRI: Magnetic resonance imaging
PTA: Puretone audiometry
SNHL: Sensorineural hearing loss
